# No NLRP3 inflammasome activity in kidney epithelial cells, not even when the NLRP3-A350V Muckle-Wells variant is expressed in podocytes of diabetic mice

**DOI:** 10.3389/fimmu.2023.1230050

**Published:** 2023-08-23

**Authors:** Sophie Carina Kunte, Julian A. Marschner, Martin Klaus, Tâmisa Honda, Chenyu Li, Manga Motrapu, Christoph Walz, Maria Lucia Angelotti, Giulia Antonelli, Maria Elena Melica, Letizia De Chiara, Roberto Semeraro, Peter J. Nelson, Hans-Joachim Anders

**Affiliations:** ^1^ Nephrologisches Zentrum, Medizinische Klinik und Poliklinik IV, Klinikum der Universität München, LMU München, Munich, Germany; ^2^ Pathologisches Institut, Medizinische Fakultät, LMU München, Munich, Germany; ^3^ Department of Experimental and Biomedical Sciences “Mario Serio”, University of Florence, Florence, Italy; ^4^ Nephrology and Dialysis Unit, Meyer Children’s Hospital IRCCS, Florence, Italy; ^5^ Department of Experimental and Clinical Medicine, University of Florence, Florence, Italy

**Keywords:** inflammasome, inflammation, diabetes, chronic kidney disease, IL-1, proteinuria

## Abstract

**Background:**

The NLRP3 inflammasome integrates several danger signals into the activation of innate immunity and inflammation by secreting IL-1β and IL-18. Most published data relate to the NLRP3 inflammasome in immune cells, but some reports claim similar roles in parenchymal, namely epithelial, cells. For example, podocytes, epithelial cells critical for the maintenance of kidney filtration, have been reported to express NLRP3 and to release IL-β in diabetic kidney disease, contributing to filtration barrier dysfunction and kidney injury. We questioned this and hence performed independent verification experiments.

**Methods:**

We studied the expression of inflammasome components in human and mouse kidneys and human podocytes using single-cell transcriptome analysis. Human podocytes were exposed to NLRP3 inflammasome agonists *in vitro* and we induced diabetes in mice with a podocyte-specific expression of the Muckle-Wells variant of NLRP3, leading to overactivation of the Nlrp3 inflammasome (Nphs2Cre;Nlrp3^A350V^) versus wildtype controls. Phenotype analysis included deep learning-based glomerular and podocyte morphometry, tissue clearing, and STED microscopy of the glomerular filtration barrier. The Nlrp3 inflammasome was blocked by feeding ß-hydroxy-butyrate.

**Results:**

Single-cell transcriptome analysis did not support relevant NLRP3 expression in parenchymal cells of the kidney. The same applied to primary human podocytes in which NLRP3 agonists did not induce IL-1β or IL-18 secretion. Diabetes induced identical glomerulomegaly in wildtype and Nphs2Cre;Nlrp3^A350V^ mice but hyperfiltration-induced podocyte loss was attenuated and podocytes were larger in Nphs2Cre;Nlrp3^A350V^ mice, an effect reversible with feeding the NLRP3 inflammasome antagonist ß-hydroxy-butyrate. Ultrastructural analysis of the slit diaphragm was genotype-independent hence albuminuria was identical.

**Conclusion:**

Podocytes express low amounts of the NLRP3 inflammasome, if at all, and do not produce IL-1β and IL-18, not even upon introduction of the A350V Muckle-Wells NLRP3 variant and upon induction of podocyte stress. NLRP3-mediated glomerular inflammation is limited to immune cells.

## Introduction

The NLR family pyrin domain containing (NLRP) 3 inflammasome is an important transducer of numerous danger signals into the activation of caspase-1 and cleavage of pro-interleukin (IL)-1β and pro-IL-18, which produces their active forms (1). Indeed, inflammasome research started from rare periodic fever syndromes characterized by the activation of caspase-1 and overexpression of IL-1β (2). Starting from its first description in the monocytic cell line THP-1 (3), the last two decades have seen an immense expansion of knowledge about the NLRP3 inflammasome in host defense as well as in sterile inflammation (1, 4). Systemic and local inflammation in metabolic disorders are especially associated with inflammasome activity, for example, in gouty arthritis (5, 6), in atherosclerosis (7), in non-alcoholic steatohepatitis (8, 9), and in diabetes (1, 10–12). Most reports on the NLRP3 inflammasome refer to its formation in immune cells, mostly myeloid cells (1, 3, 6). Indeed, the Human Protein Atlas reports NLRP3 mRNA expression exclusively in immune cells and some expression in Schwann cells (https://www.proteinatlas.org/ENSG00000162711-NLRP3/single+cell+type). Nevertheless, numerous publications report functional NLRP3 activity in non-immune cells, even if such data is not supported by publicly available transcript expression profiles. The Human Protein Atlas specifies NLRP3 mRNA expression in the human skin exclusively in immune cell subsets and not in keratinocytes (https://www.proteinatlas.org/ENSG00000162711-NLRP3/single+cell+type) but Dai, et al. reported human keratinocytes to sense dsRNA *via* the NLRP3 inflammasome leading to IL-1β release (13). NLRP3 transcripts seem absent in hepatocytes (https://www.proteinatlas.org/ENSG00000162711-NLRP3/single+cell+type/liver) but Lebeaupin and others reported NLRP3 protein expression in human hepatocytes and provided functional mouse studies consistent with hepatocyte NLRP3-mediated IL-1β release and pyroptosis (9, 14). The kidney is another epithelial organ, and most published data on the NLRP3 inflammasome relates to resident and infiltrating immune cells (15–18). The Human Protein Atlas documents the absence of NLRP3 transcripts in epithelial cells of the human kidney (https://www.proteinatlas.org/ENSG00000162711-NLRP3/single+cell+type/kidney), however, only tubular epithelial cells were captured. Shahzad, et al. reported a lower susceptibility of mice to diabetic kidney disease of Nlrp3-deficient mice transplanted with wildtype bone marrow (19). In a later study, the same authors showed that selective depletion or overexpression of the NLRP3 inflammasome in podocytes, the epithelial cells of the glomerular filtration barrier, led to protection or aggravation of diabetic kidney disease, respectively (10). These data are not only exciting from the perspective of diabetes complications but even more from the general paradigm that the NLRP3 inflammasome is only active in tissue-resident and infiltrating immune cells and not in parenchymal cells, namely, cells of epithelial lineages. Indeed, numerous studies claim NLRP3 activity in cultured podocytes or *in vivo*, even without employing convincing podocyte-specific experimental tools that would allow such a conclusion (20–27), but functional NLRP3 inflammasome in parenchymal cells is, nonetheless, summarized in focused reviews and commentaries (28, 29). We, therefore, sought to independently verify these data and performed our own single-cell transcriptome studies on kidney cell samples as well as *in vitro* experiments and *in vivo* studies with transgenic mice.

## Methods

### In silico studies

#### Single-cell RNA sequencing studies in humans

**Figure 1:**
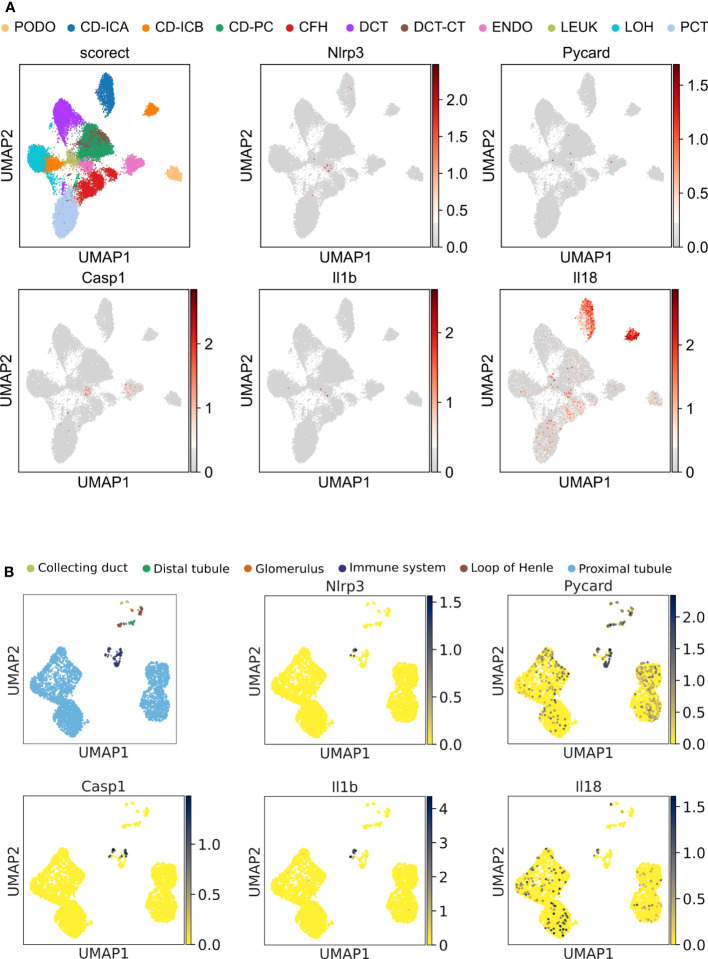
Unbiased single-cell RNA sequencing of diabetic human and healthy murine kidneys indicates the absence of canonical NLRP3 inflammasome. **(A)** UMAP plot (upper left panel) and superimposed expression pattern of Nlrp3, Pycard, Casp1, IL1b, and IL18 of single nucleus RNA sequencing data from diabetic human kidneys (GSE131882). **(B)** Same analytic procedure as in **(A)**, but for healthy C57BL/6N mice (GSE212273). PCT, proximal convoluted tubule; CFH, complement factor H; LOH, loop of Henle; DCT, distal convoluted tubule; CT, connecting tubule; CD, collecting duct; PC, principal cell; IC, intercalated cell; PODO, podocyte; ENDO, endothelium; MES, mesangial cell; and LEUK, leukocyte.

**Figure 2:**
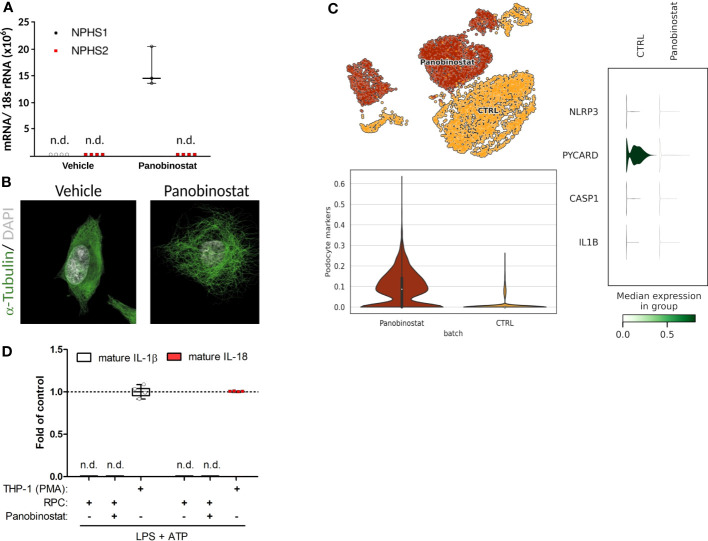
Primary human podocyte-like cells do not indicate complete functionality of canonical NLRP3 inflammasome. **(A)** Normalized NPHS1 and NPHS2 mRNA expression in primary human renal progenitor cells (hRPCs) after 48 h of vehicle or panobinostat treatment. **(B)** Confocal imaging of α-tubulin (green) and DAPI (white) stained vehicle and panobinostat treated hRPCs. **(C)** UMAP clustering for unbiased single-cell RNA sequencing data from vehicle (CTRL) or panobinostat-treated hRPCs (upper left panel, GSE195797). NLRP3, PYCARD, CASP1, and IL1B expression per UMAP cluster. **(D)** ELISAs of supernatants from panobinostat-treated hRPCs do not detect cleaved IL-1β and IL-18, respectively, after stimulation in contrast to phorbol myristate acetate treated THP-1 cells; n = 3, n.d., not detectable.

Unbiased single-nucleus RNA sequencing data (snRNA-seq) on cryopreserved human diabetic kidney samples were obtained from NCBI, GEO accession number GSE131882. The data were processed using the Scanpy (30) pipeline. Genes expressed in >3 nuclei and nuclei with at least 500 genes were retained. The normalization of the data was conducted using the Scran (31) package, which included assuming equal size factors, normalizing the library size to counts per million, and log-transforming the count data. The Harmony (32) integration pipeline was employed to reduce data dimensions and remove batch effects. The 3,000 most variable genes were used for the principal component analysis. The Uniform Manifold Approximation and Projection (UMAP) was applied for unsupervised clustering based on the first 50 integrated principal components. Cell annotation was completed manually based on mRNA-marker expression profiles from the original article (33). The results of this re-analysis are shown in **Figure 1A**. Data shown in **Figure 2C** are derived from a reanalyzed and previously published scRNAseq dataset (GSE195797) containing data generated on human RPCs treated with vehicle or panobinostat (34). We handled the dataset in the Scanpy environment and evaluated the expression of the genes of interest, as shown in **Figure 2C**.

#### Single-cell RNA sequencing studies in mice

A previously published dataset (GSE212273) containing single-cell data generated from healthy male mouse kidney (35) was reanalyzed to investigate the role of the NLRP3 inflammasome in the different kidney cell types. As the dataset (GSE212273) integrates samples from three experimental points t0, t2, and t30, we loaded the dataset using the Scanpy framework, removed the t2 and t30 samples, and clustered and annotated the data after recalculating the neighborhood graph on the latent space. After filtering, we obtained 3,766 cells with less than 40% of mitochondrial read rate and expressing more than 200 genes. The scran computeSumFactors method, which implements the deconvolution strategy for scaling normalization, was used to normalize cell-specific biases. After the addition of a pseudocount of 1, all counts were log-transformed. As a next step, we kept only “informative” genes and those were further used for dimensional reduction by principal component analysis. A neighborhood graph of observations was constructed using the first 50 principal components. Therefore, the pp.neighbors function, which estimates the connectivity of data points (Uniform Manifold Approximation and Projection (UMAP) algorithm), was used. To annotate the clusters we ran the tl.rank_gene_groups function (Wilcoxon rank-sum method) to define the marker genes of each cluster as previously shown (35). The results of this reanalysis are shown in **Figure 1B**.

### In vitro studies

#### Cell isolation and culture of primary human renal progenitor cells

Human renal progenitor cells (hRPC) were obtained and cultured as described in a previous study (36). The cells were used in passages 1-3 and cultured in EGM™-MV medium + 20% FCS (Hyclone™ Defined Fetal Bovine Serum, Cytiva) under sterile conditions at 37°C and 5% CO_2_ (the culture medium recipe can be found in **Supplementary Table 1**). To differentiate hRPCs into podocyte-like cells, hRPCs were cultured in Endothelial Cell Growth Medium (ECGM) + FCS. At 80% to 100% cell confluence, the medium was changed to ECGM without FCS for 24 hours. Cells were then incubated using filtered 100 ml ddH_2_O, 1.56 g Dulbecco’s Modified Eagle´s Medium/Nutrient Mixture F-12 Ham (DMEM-F12), 0.12 g sodium-bicarbonate, and 0.1 µM panobinostat. After 48 hours of incubation, the differentiation to podocyte-like cells was completed.

#### THP-1 cell culture

Human-derived THP-1 (DSMZ# ACC16) were cultivated in RPMI medium 1640 with GlutaMAX^TM-I^ (Gibco) enriched with 20% FCS and 100 µM penicillin-streptomycin. Differentiation into a macrophage-like phenotype was achieved by 72h treatment with 50 ng/mL phorbol myristate acetate (PMA).

#### NLRP3 inflammasome activation

Native RPCs, panobinostat-treated RPCs, and PMA-treated THP-1 cells were stimulated with 100 ng/mL of lipopolysaccharides (LPS, Sigma Aldrich) for 3h in their respective (differentiation) media. Subsequently, the media were aspirated, and cells were stimulated with 5 mM of Adenosintriphosphate (ATP, Sigma Aldrich). Media were harvested, and secreted IL-1β and IL-18 were detected by ELISA (#88-7261-88, #BMS267INST, Invitrogen), according to the manufacturer’s protocol.

#### RNA isolation and qPCR

Total RNA was extracted from hRPC cultures using the pure Link RNA Mini Kit and samples were preserved in RNAlater, according to the manufacturer’s instructions. A total of 2 µg RNA was used to transcribe cDNA by reverse transcription polymerase chain reaction (PCR) using the reagents listed in **Supplementary Table 2**. For each sample, the reaction was run both in the presence and absence of reverse transcriptase in order to adjust for residual genomic DNA contamination during qPCR. Using a Mastercycler Pro (Eppendorf, Germany), first a denaturation was performed at 65°C for 10 minutes, followed by reverse transcription at 42°C for 90 minutes. For quantitative real-time PCR (qPCR) from cDNA, the reagents listed in **Supplementary Table 2** and primers listed in **Supplementary Table 3** were used. As we used 18s rRNA as a reference transcript for relative quantification, qPCR data for genes of interest were normalized to 18s. Controls (ddH2O) were negative for target and reference genes. Each amplification step included the following: initiation phase at 95°C, annealing phase at 60°C, and amplification phase at 72°C. This procedure was repeated for 40 cycles (37). We designed cDNA-specific primers that targeted the most CCDS-approved transcripts. All samples not exceeding background fluorescence were classified as undetectable. Melting curve profiles of every sample were analyzed to identify unspecific products and primer dimers. If necessary, agarose gels for further verification of PCR products were used.

### In vivo studies

#### Animals

*Nphs2 Cre; Nlrp3^WT/A350V^* (Podo*-Cre; Nlrp3^WT/A350V^*) mice in the C57BL/6J background were generated by crossing *Nphs2 Cre* mice (B6.Cg-Tg(NPHS2-cre)295^Lbh^/J (38), Jax strain ID #008205) with *Nlrp3^WT/A350V^* mice (B6.129-Nlrp3^tm1Hhf^/J (2), Jax strain ID #017969 (39), **Supplementary Figure 1A**). We used littermate mice for all experiments (**Supplementary Figure 1B**). Nlrp3 KO mice were provided by J. Tschopp (University of Lausanne, Lausanne, Switzerland) and by V. Dixit (Genentech, San Francisco, California, USA). We co-housed male or female mice from 3 weeks of age onwards, in groups of 4-5 in cages under specific-pathogen-free conditions with unlimited access to standard chow (ssniff, Germany) and desalinated water. We sterilized cages, nestlets, food, and water by autoclaving before use. All experimental procedures were approved by the local government authorities according to the European equivalent of the NIH’s Guide for the Care and Use of Laboratory Animals (directive 2010/63/EU).

#### Isolation of glomeruli and end point PCR

Paramagnetic isolation of glomeruli from both genotypes was performed as described in a previous study (40). Genomic DNA was isolated from harvested glomeruli by the addition of Direct PCR Tail Peqgold (Peqlab) and proteinase K (Qiagen), according to the manufacturer’s protocol, and incubation for 3h at 55°C and 800 rpm. Subsequent inactivation of proteinase K was achieved by heating the sample to 85°C for 45 min. End point PCR using Taq DNA Polymerase (M0273X, New England Biolabs) and primers indicated by The Jackson Laboratories for the B6.Cg-Tg(NPHS2-cre)295^Lbh^/J strain verified successful excision of the floxed transgene by Nphs2-driven Cre recombinase (**Supplementary Figure 1D**).

#### Study design

**Figure 3:**
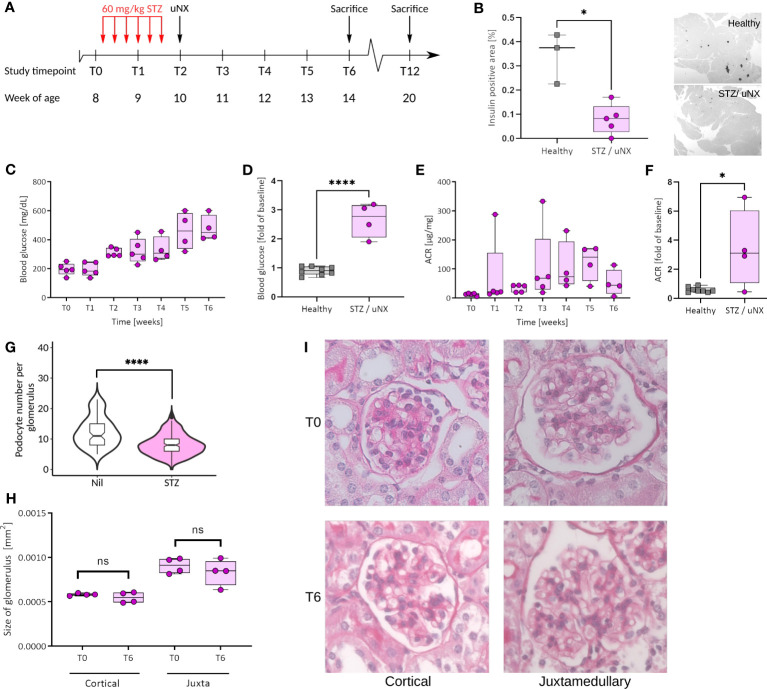
Female C57BL/6J mice respond with loss of β-cells, hyperglycemia, and albuminuria to streptozotocin (STZ) treatment and uninephrectomy (uNX). **(A)** Female C57BL6/J mice were injected with STZ (six intraperitoneal injections of 60 mg/kg BW every other day prior to uNX and followed for 4 and 10 weeks). **(B)** Immunohistochemistry for insulin in pancreatic tissue of control and STZ/uNX treated mice at T6. Time course **(C, E)** and T6 **(D, F)** of blood glucose **(C, D)** and albumin creatinine ratio (ACR; **E, F**) of STZ/uNX treated mice or control and STZ/uNX treated mice, respectively. **(G)** Podocyte number per glomerulus at T6. **(H, I)** Morphometric determination of the glomerular size of STZ/uNX treated mice from Periodic acid-Schiff (PAS) stained paraffin sections at indicated time points. n = 5, *p<0.05, ****p < 0.0001. ns, not significant.

**Figure 4:**
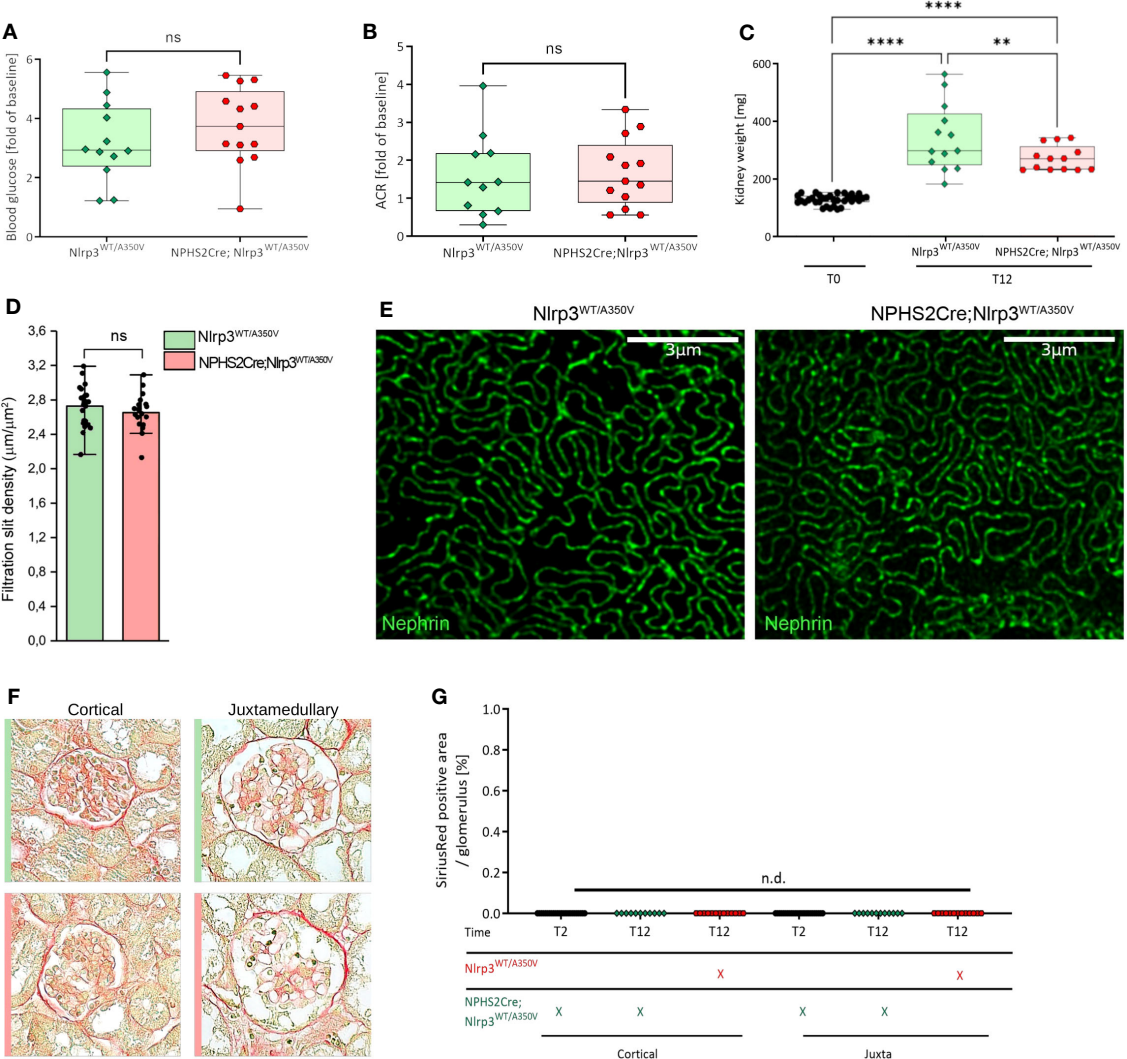
Introduction of a podocyte-specific Nlrp3^WT/A350V^ mutation does not aggravate the phenotype after STZ/uNX treatment. Female Nlrp3^WT/A350V^ and Podo-Cre; Nlrp3^WT/A350V^ mice underwent treatment indicated in **Figure 4A**. Blood glucose **(A)**, ACR **(B)**, and kidney weight **(C)** were determined at T12 (n ≥ 11). **(D, E)** Density of the glomerular slit diaphragm was investigated using stimulated emission depletion microscopy (STED) for nephrin-stained kidney sections of either genotype at T12 (n = 3). **(F, G)** Representative sirius red staining pictures quantified in **(F)** for signs of glomerulosclerosis.**p<0.01, ****p < 0.0001, n.d., not detectable. ns, not significant.

**Figure 5:**
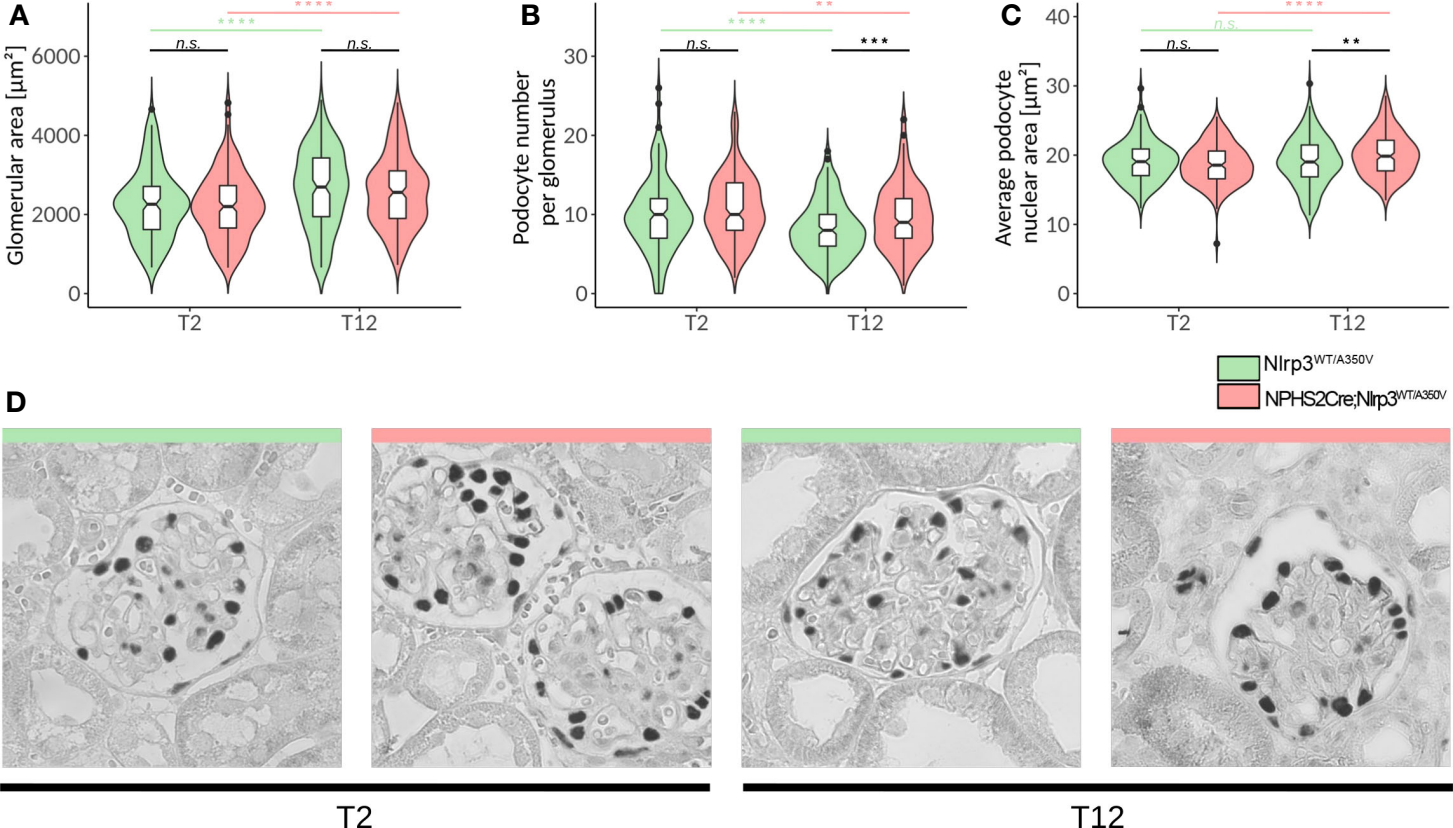
Deep learning-assisted morphometrics reveal podocyte hypertrophy in Podo-Cre; Nlrp3^WT/A350V^ mice. A U-Net image segmentation algorithm was used to segment glomeruli and podocyte nuclei in WT-1-stained images **(D)** and calculate the glomerular area **(A)**, average podocyte number per glomerulus **(B)**, and average podocyte nuclear area **(C)**. **(D)** Representative images of glomeruli from WT-1-stained kidney sections of female Nlrp3^WT/A350V^ and Podo-Cre; Nlrp3^WT/A350V^ mice after STZ and uNX at different time points. n ≥ 11, **p<0.01, ***p<0.001, ****p < 0.0001. n.s., not significant.

**Figure 6:**
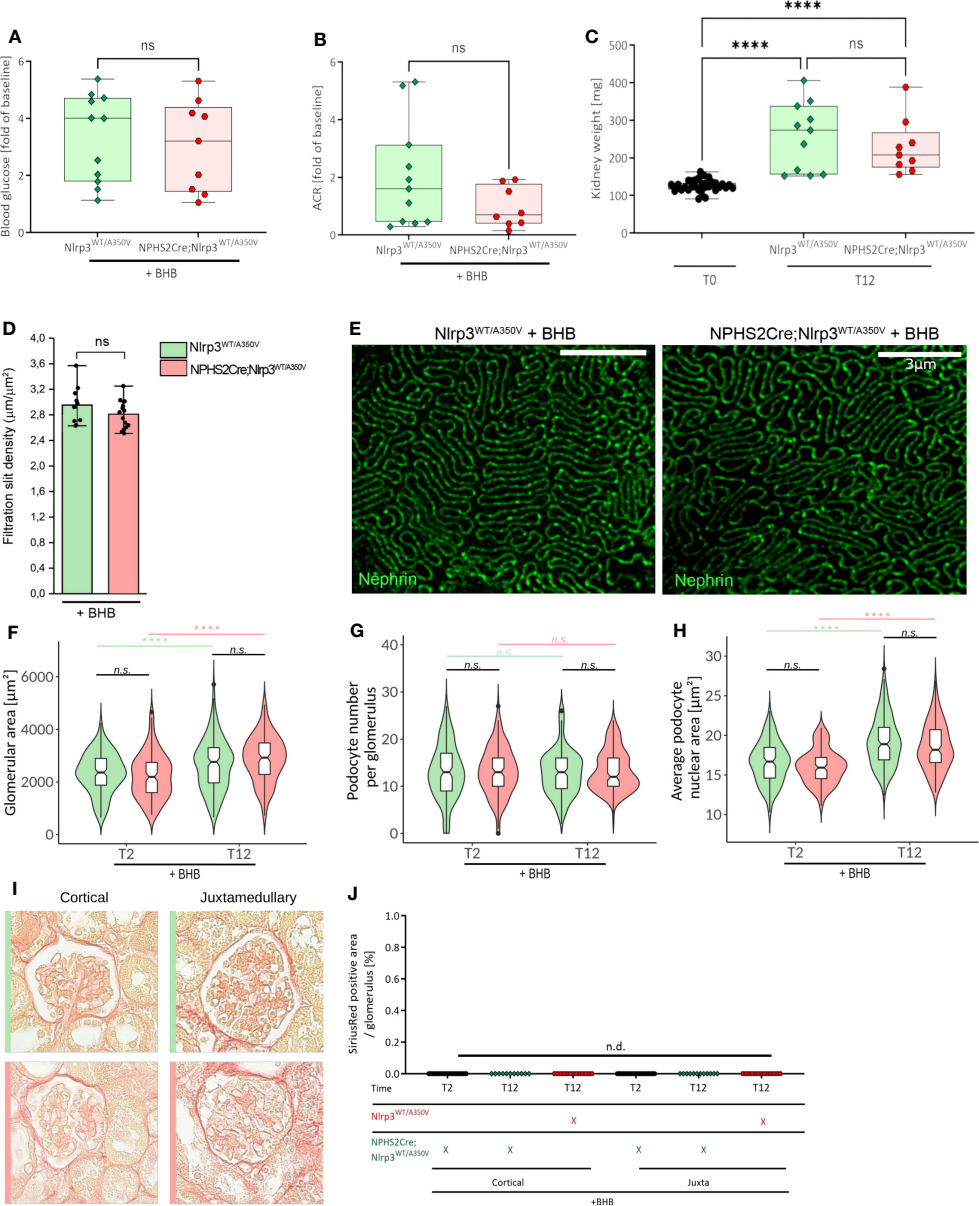
Treatment of Podo-Cre; Nlrp3^WT/A350V^ mice with β-hydroxybutyrate *(BHB) does rescue the Nlrp3^WT/A350V^ induced phenotype.* Female Nlrp3^WT/A350V^ and Podo-Cre; Nlrp3^WT/A350V^ mice underwent treatment indicated in **Figure 3A** and received BHB-enriched food ad libitum between T3 and T12. Blood glucose **(A)**, ACR **(B)**, and kidney weight **(C)** were determined at T12 (n ≥ 11). **(D, E)** Density of the glomerular slit diaphragm was investigated using stimulated emission depletion microscopy (STED) for nephrin-stained kidney sections of either genotype at T12 (n = 3). Deep learning assisted morphometrics to calculate the glomerular area **(F)**, average podocyte number per glomerulus **(G)**, and average podocyte nuclear area **(H)** from WT-1-stained kidney sections as shown in **Figure 5**. Representative images of sirius red stained sections **(I)** quantified in **(J)** for signs of glomerulosclerosis. (n ≥ 11), ****p < 0.0001. ns, not significant.

We based group size calculation on numeric assumptions derived from our pilot studies (**Supplementary Figure 2**). The total numbers of animals were 5 per group for the studies presented in **Figure 3** and **Supplementary Figure 2** (C57BL/6J wildtype, STZ/uNX treatment), and 11-13 per group for the studies presented in **Figures 4**–**6**. We collected data for each treatment group from one (**Figure 3** and **Supplementary Figure 2**) or at least seven independent experiments (**Figures 4**–**6**).

#### Drug interventions

We injected female C57BL/6J mice at 8 weeks of age (Charles River, Sulzfeld, Germany) intraperitoneally with 10 mg/kg/day oridonin (Cayman/Biomol, 25665) for 14 consecutive days, starting 1 week after uninephrectomy. Control animals of the same background, age, and sex received 10% DMSO/90% PBS (vehicle) at the same volume and time points. To treat either transgenic or wildtype animals with β-hydroxybutyrate (BHB), a BHB precursor, 1,3-butanediol (Sigma Aldrich, Taufkirchen, Germany), was added to 20% mass fraction of standard chow and supplied ad libitum.

#### Uninephrectomy

We conducted uninephrectomy in animals at 10 weeks of age as described in a previous study (41). Briefly, anesthesia included a mixture of medetomidine, midazolam, and fentanyl to achieve analgesia, amnesia, and hypnosis prior to surgery. Animals were heated during all phases of surgery as described in a previous study (42). Core body temperature maintenance between 36.5 and 38.5°C during the procedure was supervised by using online rectal temperature recording probes. Fully anesthetized animals received a left flank incision of 1 cm in length. The kidney was decapsulated and the adrenal gland was preserved before ligating the renal pedicle using non-absorbable sutures. After resection of the kidney, wounds were closed using absorbable sutures for the peritoneal and cutaneous layers. Buprenorphine pain control was started 30 min prior to the antagonization of narcosis. Dosages and treatment regimens of narcosis, antagonization, and analgesia are listed in **Supplementary Table 4**. For adjustment of fluid losses, we administered 200 µL of saline into the peritoneal cavity. We handled sham-operated mice in the same manner, except for pedicle ligation and kidney resection.

#### Blood glucose

Blood was collected at the indicated time points. Blood glucose was determined using an Accu Chek blood glucometer (range 11-600 mg/dL; Roche, Mannheim).

#### Murine albumin sandwich ELISA

Urine was collected at predetermined time points, centrifuged at 8000g and 4°C for 6 min, and stored at -80°C until analysis. Reagents, working standards, and diluted samples (dilution depending on the respective blood glucose: < 200 mg/dL: 1:150; 200-350 mg/dL: 1:250; >350 mg/dL: 1:500) were prepared according to the manufacturer’s instructions (Mouse Albumin ELISA Quantitation Set, Bethyl Laboratories). Measurements of absorption (450 nm) were performed using a photometer (GENios Plus, Tecan Austria GmbH). Mean values were built from duplicates and the blank was subtracted. Albumin values (µg/dL) were calculated using a standard curve.

#### Blood urea nitrogen

At the indicated time points, plasma was collected in the presence of heparin, centrifuged at 8000 g and 4°C for 6 min, and stored at -80°C until analysis. Reagents, working standards, and undiluted samples were prepared according to the manufacturer’s instructions (Urea FS, DiaSys Diagnostic Systems GmbH). Measurements of absorption (340 nm) were performed after 60 and 120 seconds using a photometer (GENios Plus, Tecan Austria GmbH). Values were calculated as follows: BUN=((value_60s_-value_120s_)-Blank) * 0.467. Blood urea nitrogen values (mg/dL) were determined using linear regression.

#### Creatinine

Urine was collected at predetermined time points, centrifuged at 8000g and 4°C for 6 min, and stored at -80°C until analysis. Reagents, working standards, and diluted samples (1:9) were prepared according to the manufacturer’s instructions (Creatinine FS, DiaSys Diagnostic Systems GmbH). Measurements of absorption (492 nm) were performed after 60 and 120 seconds using a photometer (GENios Plus, Tecan Austria GmbH). Values were calculated as follows: CREA=((value_120s_-value_60s_)-Blank). Creatinine values (mg/dL) were determined using linear regression. Albumin-Creatinine-Ratio (ACR) was determined by dividing the respective albumin value by the respective creatinine value.

#### Tissue processing staining and morphometry

Kidneys from T2, T6, and T12 were stored in 4% buffered formalin for 18-24 h, embedded in paraffin, and 2–4 µm sections were prepared for periodic acid–Schiff (PAS) and Picro-Sirius Red or immunostaining for WT-1, nephrin, and Nlrp3 by deparaffinization (xylene), rehydration (ethanol series), and blockade of endogenous peroxidase (3% H_2_O_2_ in methanol). For antigen retrieval, we used either proteinase K or microwave-heated unmasking solution at pH 6. Sections were blocked with avidin and biotin for 20 min each prior to staining. After the slides were washed in PBS, they were incubated with the primary and secondary antibodies as indicated in **Supplementary Table 5**. Low-power field (40x) images were taken with a Leica DMRBE Research Microscope for each staining. Sections were analyzed as follows: *PAS:* Glomerular hypertrophy was determined by analyzing the size of both 10 randomly selected cortical and juxtamedullary glomeruli. *Sirius Red:* Both 10 cortical and juxtamedullary glomeruli were analyzed regarding glomerulosclerosis as described in a previous study (41). The positive signal was measured in pixels and normalized to the whole glomerular area section using ImageJ 1.53f52 (43). *Nlrp3:* Sections were stained with Nlrp3 for identification of the inflammasome complex. Nlrp3-positive cells were quantified in 10 randomly selected glomeruli. The positive signal was measured in pixels and normalized to the whole glomerular area section using ImageJ 1.53f52 (43).

#### Deep learning-based podocyte morphometry

To further elucidate differences between genotypes at the cellular level, we performed quantitative image analysis using a U-Net deep learning segmentation algorithm. To analyze podocyte morphometry, we used WT-1-stained slides specific for podocyte nuclei and nephrin-stained slides highlighting the podocyte cytoplasm. Glomeruli were imaged and saved in 2048 x 2048-pixel frames, at a resolution of 8.8 pixels per micron. Glomeruli (WT-1 and nephrin staining) and podocyte nuclei (only WT-1 staining) in 400 images were manually annotated to train and validate the algorithm. Subsequently, we implemented a deep learning algorithm inspired by U-Net (44). To ensure decent segmentation quality, we used data augmentation and weight map techniques (45). The U-net was then trained separately on each task (glomerular tuft or podocyte nuclei) and segmentation quality was evaluated with validation subsets that were left out during training time. Finally, segmentations were post-processed to exclude non-representative structures, such as glomeruli, that were cut at an edge or merged structures. To obtain the nephrin area, the Otsu threshold filter was used on the glomerular tuft segmentations (46). Finally, the processed segmentations were used to automatically compute morphometrics (47, 48). These included glomerular tuft area, number of podocytes, average podocyte nuclear area, area density of podocytes, ratio podocyte nuclei/glomerular tuft, podocyte cytoplasm area, and ratio of podocyte cytoplasm/glomerular tuft. Additionally, the 2-dimensional readouts were extrapolated into 3d using model-based stereology (49–51). Using this, the volume of the glomerular tuft, volume density of podocytes, and total count of podocytes in the whole glomerulus were estimated. Finally, statistical morphometry analysis was performed with R.

#### Optical tissue clearing

Optical clearing of the kidneys was employed to quantify filtration slit density. Therefore, kidneys were dissected and incubated at 4°C in a hydrogel solution (4% v/v acrylamide, 0.025% v/v bisacrylamide, 0.25% w/v VA-044 initiator, 4% PFA) for 24 hours. The gel was polymerized at 37°C for 3 h, and oxygen within the tubes was minimized by filling the hydrogel solution to the top. The samples were removed from the hydrogel solution, cleared (200 mmol/l boric acid, 4% SDS, pH 8.5), and incubated at 50°C for 24 hours. Kidneys were dissected in 300 µm thick slices (Vibratome) and incubated at 50°C for 5 days. The clearing solution was replaced every day. Then, samples were incubated in PBST (0.1% Triton-X in 1 × PBS) for 24 hours. Immunolabeling was conducted as follows: samples were incubated in primary antibody (anti-NPHS1, R&D, Cat# AF4269, RRID: AB_2154851) at 37°C for 24 h, washed in PBST at 37°C for 4 h, incubated in secondary antibody (donkey anti-sheep IgG-TRITC, Thermo Fisher Scientific, Cat# A24566, RRID: AB_2536034) at 37°C for 24 h, and washed at 37°C for 4 h. Dilutions were made using PBST in all steps. Samples were mounted in 80,2% fructose and 0.5% (v/v) 1-thioglycerol.

#### Stimulated emission depletion microscopy and slit diaphragm density quantification

An SP8 STED 3X confocal microscope (Leica Microsystems) was used to take STED xyz images (i.e., z-stacks acquired along 3 directions: x, y, and z axes). The TRITC-labeled secondary antibody was exited at 555 nm with a white light laser. The respective emission was recorded from 565 to 605 nm. The 660 nm pulsed-depletion laser had a gating of 0.7 to 6 ns. Images were captured with a Leica HC PL APO CS2 100x/1.40 oil STED White objective. The images were further analyzed using Huygens Professional software version 18.04. Z stacks of the NPHS1 signal were acquired (at least 5 μm thick) to quantify the filtration slit coverage. Thus, all images of each z stack were merged. The foot process length as well as the area of the field were manually plotted and analyzed with Image J. The total length of the foot processes was divided by the total area of interest. An evaluation was conducted for five randomly selected areas of each glomerulus of at least five glomeruli per mouse.

### Statistical analysis

We either report data as individual measurements with superimposed plunger bar plots or violin plots. Prior to every other statistical analysis, we tested for normal distribution (Anderson-Darling and Shapiro-Wilk test), homoscedasticity (Brown-Forsythe test), and outliers (Grubb’s test). Normally distributed and homoscedastic data sets were tested for statistically significant differences *via* ANOVA and *post-hoc* Bonferroni’s correction for multiple comparisons. We corrected heteroscedastic data following Games-Howell’s *post-hoc* test. We compared not normally distributed data sets using Kruskal-Wallis and Wilcoxon-Mann-Whitney-U testing. We considered a value of p < 0.05 to indicate statistical significance. P-values were indicated as p > 0.05 n.s., p < 0.05 ∗, p < 0.01 ∗∗, p < 0.001 ∗∗∗, and p < 0.0001 ∗∗∗∗.

## Results

### Expression of NLRP3 inflammasome-related transcripts in the kidney

The Human Protein Atlas scRNAseq data set displayed *NLRP3* transcripts only in intrarenal immune cells but not in epithelial cells, although the samples analyzed did not include podocytes (https://www.proteinatlas.org/ENSG00000162711-NLRP3/single+cell+type/kidney). We, therefore, analyzed another dataset from a human diabetic kidney with a clear podocyte cluster. In podocytes and all other kidney epithelial cells, *NLRP3, PYCARD, CASP1*, *IL1B, and IL18* transcripts were all absent (**Figure 1A**). Transcripts of the three genes, whose products make up the NLRP3 inflammasome, were only sparsely detected in the leukocyte and endothelial cell cluster. Published data on the NLRP3 inflammasome used mouse models, hence we performed unbiased RNA sequencing in C57BL/6J mice and identified glomerular cells based on the expression of *Nphs1*, *Podxl*, *Cldn1*, and *Peacam1* (**Supplementary Figure 3**). Superimposing the expression levels of canonical NLRP3 inflammasome genes *Nlrp3*, *Pycard*, *Casp1*, *IL1b*, and *IL18* on the Uniform Manifold Approximation and Projection (UMAP)-generated clusters confirmed the absence of all investigated transcripts, except for *Pycard* (**Figure 1B**). The expression of *Nlrp3* was entirely restricted to cells of the immune system. Thus, in human and mouse kidneys, NLRP3 transcripts are exclusively detectable in immune cells and not in podocytes.

### Human podocytes cannot be stimulated to secrete IL-1β or IL-18

Published data on NLRP3 inflammasome formation and IL-1β secretion in podocytes are based on immortalized podocyte cell lines or primary podocytes, which are both a concern, because a key characteristic of podocytes is their postmitotic state that is incompatible with cell division or expansion in culture (52). Indeed, fully differentiated podocytes engage their actin cytoskeleton to maintain their foot processes, which is incompatible with forming a mitotic spindle and undergoing cell division without ending in mitotic catastrophe (53). As a more reliable *in vitro* model of podocyte biology, we generated human podocytes *via* differentiation from immature human podocyte progenitors, which are easy to maintain and expand in culture, before initiating the differentiation process into mature and postmitotic podocytes (**Figure 2**) (36, 54). Strong mRNA induction of the filtration slit protein nephrin/NPHS1 and the characteristic morphological features indicated terminal differentiation into human podocytes (**Figures 2A, B**). Unbiased mRNA sequencing of such cultures confirmed the expression of numerous markers of podocyte differentiation but the lack of *NLRP3*, *PYCARD*, *CASP1*, and *IL1b* (**Figure 2C**). Exposing such human podocytes to the two signals required to induce canonical NLRP3 inflammasome signaling, i.e., lipopolysaccharides (LPS) and adenosine triphosphate (ATP), did not induce the secretion of cleaved IL-1β or of IL-18 (**Figure 2D**). We conclude that human podocytes lack the capacity for canonical NLRP3 inflammasome function, i.e., secretion of IL-1β and IL-18, because they also do not express enough of the necessary components.

### Transgenic overactivation of Nlrp3 activity by introducing the Muckle-Wells Syndrome gene variant into podocytes and induction of diabetes

Mice carrying the Muckle-Wells Syndrome gene variant A350V develop Muckle-Wells syndrome-like systemic inflammation due to overactivation of the NLRP3 inflammasome and IL-1β production (39). Restricting the expression of the A350V variant to macrophages is sufficient to reproduce this phenotype (55), indicating that these cells are the major source of inflammasome activation and IL-1β secretion. To ultimately exclude the possibility that podocytes could form a functional NLRP3 inflammasome and produce IL-1β promoting glomerular injury, we generated Podo*-Cre; Nlrp3^WT/A350V^* and *Nlrp3^WT/A350V^* littermates (**Supplementary Figure 1B**). Our mice bred at Mendelian ratios and did not show any spontaneous phenotype. To induce significant hemodynamic and metabolic podocyte stress, we followed a dual approach, i.e., to a) increase glomerular hyperfiltration by reducing total filtration surface *via* uninephrectomy (uNX) and b) to induce hyperglycemia with STZ, which deactivates the tubuloglomerular feedback and leads to further hyperfiltration of the remaining glomeruli. After testing several STZ regimens in both sexes (**Supplementary Figure 2**), we selected 60 mg/kg injected on six alternate days intraperitoneally on female mice (**Figure 3A**). This treatment induced loss of pancreatic β-cells and consistent hyperglycemia (**Figures 3B–D**). STZ and uNX induced albuminuria (**Figures 3E, F**) and a sign of loss of podocytes (**Figure 3G**), while at this early stage of the disease, blood urea nitrogen (BUN, **Supplementary Figure 5A**) and glomerular size remained unaltered (**Figures 3H, I**). uNX and STZ injection in Podo*-Cre; Nlrp3^WT/A350V^* and *Nlrp3^WT/A350V^* littermates induced the same levels of hyperglycemia (**Figure 4A**), albuminuria (**Figure 4B**), and integrity of the slit diaphragm (**Figures 4D, E**). Interestingly, kidney hypertrophy was less pronounced in Podo*-Cre; Nlrp3^WT/A350V^* animals (**Figure 4C**). The glomeruli of the Podo*-Cre; Nlrp3^WT/A350V^* strain did not show any signs of glomerulosclerosis, as indicated by sirius red stainings (**Figures 4F, G**). These results strengthen the hypothesis that podocytes do not involve the NLRP3 inflammasome in the context of hyperfiltration-induced glomerular injury, even with a gain-of-function mutation.

### Deep learning-assisted podocyte morphometrics indicate a non-canonical role of overactive NLRP3 in podocytes

To exclude further sources of analytical bias, we employed a machine learning algorithm on WT-1 and nephrin-stained kidney sections that were previously used to conduct in-depth podocyte morphometry (**Supplementary Figure 4**) (56). Indeed, the glomerular area increased significantly from T2 (uNX) to T12, but without a difference between the genotypes (**Figure 5A**). In contrast, cell counts and nuclear area were both significantly increased in podocytes carrying an active *Nlrp3^A350V^* allele (**Figures 5B, C**). These results surprisingly indicate, that if anything, the gain-of-function in Nlrp3 may lead to a higher resilience of the podocytes in response to hyperfiltration stress. Therefore, podocyte morphometrics rather suggests a better resilience of A350V podocytes to hemodynamic and metabolic stress but no sign of inflammation or injury compatible with canonical NLRP3 function as reported.

### β-Hydroxybutyrate reverses podocyte changes in Nphs2-Cre; Nlrp3^WT/A350V^ mice

To verify that podocyte hypertrophy is an NLRP3-mediated effect, we supplemented animal chow with 1,3-butanediol, a precursor of β-hydroxybutyrate (BHB) and NLRP3 inhibitor (57), with otherwise unchanged experimental conditions. BHB treatment did not alter hyperglycemia, albuminuria, BUN, and slit diaphragm integrity (**Figures 6A, B, D, E**, **Supplementary Figure 5C**) but reversed the podocyte changes observed in Podo*-Cre; Nlrp3^WT/A350V^* mice (**Figure 6C**), i.e., higher podocyte counts per glomerulus and greater podocyte nuclear area (**Figures 6G, H**). Notably, the overall increase in glomerular size was not affected by BHB treatment (**Figure 6F**) and glomerulosclerosis was still not detectable (**Figures 6I, J**).

## Discussion

We had intended to clarify the discrepancy between scRNAseq data released by the Human Protein Atlas and an impressive number of publications on the presence and function of the NLRP3 inflammasome in epithelial cells of the kidney, e.g., under diabetic conditions (1, 10–12). Our data analysis from single-cell transcriptomics of human diabetic kidney, healthy mouse kidney, and human differentiated and postmitotic podocytes verify the absence of relevant amounts of transcripts for NLRP3, IL-1β, and other related molecules. To exclude the possibility of a sensitivity issue, we generated transgenic mice expressing the A350V variant of NLRP3 selectively in podocytes, which is known to lead to an overactivation of the NLRP3 inflammasome with increased IL1-β release in mouse macrophages (39). A careful phenotype analysis could exclude that the gain-of-function mutation in podocytes was followed by an aggravated pathology under diabetic conditions and additional podocyte stress induced by uninephrectomy. Our results independently validate the data released by the Human Protein Atlas in terms of NLRP3 expression in podocytes and point to the same conclusions regarding mouse kidneys, further questioning studies that report and conclude otherwise.

Single-cell transcriptome analysis has become a powerful tool to determine cell type-specific expression patterns. However, the quality of the analysis depends on sample quality and processing, way of sequencing, and arbitrary parameters. For example, the dataset released by the Human Protein Atlas lacks a podocyte cluster, probably because podocytes are few and get easily lost during kidney processing unless special enzymatic treatments provide access to the cells inside the Bowman’s capsule of the glomeruli. The amount of NLRP3 transcripts may be low under healthy conditions and may increase only upon certain stimuli, therefore we first analyzed kidneys from individuals with diabetes and “diabetic kidney disease”. Diabetes as a stimulus was of interest in this context because many publications report NLRP3 inflammasome activity in podocytes in the context of hyperglycemia and diabetes (1, 10–12). Our finding, that even in the diabetic kidney NLRP3 expression is restricted to infiltrating immune cells, contradicts such studies but is in line with the NLRP3 mRNA expression pattern in most other solid organs and the skin, according to the Human Protein Atlas. The same is true for other NLRP3 inflammasome-related transcripts such as caspase-1 and pro-IL1β. To ultimately exclude the possibility that whole kidney isolates lack sufficient sensitivity to detect NLRP3 transcripts, we generated terminally differentiated and postmitotic human podocytes from their origin progenitors, which, due to their immature status, can be expanded in culture easily. However, neither progenitors nor podocytes expressed NLRP3 inflammasome-related transcripts, which is contradictory to data derived from immortalized “podocyte” cell lines or “primary podocyte cultures”, knowing that podocytes lose all their characteristics and largely die during the isolation procedure. Because of these findings, the confidence expressed in the medical literature on a functional NLRP3 inflammasome in epithelial cells is questionable.

IL-1β release is the main functional consequence of NLRP inflammasome activation and is well documented in the various cell types of the myeloid lineage. Nevertheless, numerous studies claim IL-1β release also from epithelial cells (20–27), which would require mRNA and protein expression of pre-IL-1β in the same cell, which we could not verify. Accordingly, human podocytes could not be stimulated to secrete mature IL-1β and not even mature IL-18 using LPS/ATP, the two classical stimuli used as a positive control for NLRP3 inflammasome activation. Whether podocyte IL-1β production reported by others included immune cell contaminants is speculative. However, we can absolutely exclude this possibility from our experiments because the human podocyte progenitor cultures were obtained by negative selection for CD45 and clonal dilution, i.e., all the cultures derived from a single immature podocyte progenitor cell.

To the best of our knowledge, this is the first study to report data from Podo*-Cre; Nlrp3^WT/A350V^* mice, a genetic approach to overcome sensitivity issues with putative NLRP3 inflammasome signaling in podocytes. Shahzad, et al. reported data using homozygous Podo*-Cre; Nlrp3^A350V/A350V^* mice suggestive of NLRP3 inflammasome activation in podocytes in the context of diabetic kidney disease. We cannot explain these discrepant findings in view of glomerular pathology, but it is of note that the homozygous state of these mice eliminated Nlrp3 from all non-podocyte cells of the mice as a confounding difference to their control mice, which makes data interpretation of the phenotype difference difficult (**Supplementary Figure 1C**). The same logic applies to other studies utilizing global NLRP3-knockout mouse models to conclude on cell-specific functionality of the NLRP3 inflammasome (20). Our subtle phenotype analysis rather pointed towards minimal effects of NLRP3^A350V^ on podocyte resilience and hypertrophy, which contrast with the idea of NLRP3-mediated glomerular injury.

Our study certainly has some limitations. We did not study human podocyte progenitor transcriptomes under diabetic conditions, but the same cells did not produce IL-1β even under more potent inducers of NLRP3 mRNA expression, i.e., LPS. Within 12 weeks, our mouse model of diabetes did not develop overt diabetic nephropathy, not even upon uninephrectomy, whereas Shahzad, et al. reported an impressive degree of glomerular injury without uninephrectomy albeit after 32 weeks. Strain differences may account for this difference. However, few juvenile humans develop kidney disease within the first decade of hyperglycemic conditions because the kidney adapts well to hyperglycemia-induced hyperfiltration. We, therefore, consider our kidney phenotype in STZ-induced diabetes well in line with the kidney phenotype of human diabetes in an relatively early phase of life.

We conclude that NLRP3 is at best poorly expressed or virtually absent in podocytes as it is in the other epithelial cells of the kidney. Thus, any NLRP3 inflammasome activation in the kidney should preferentially refer to resident and infiltrating myeloid cells. We were unable to induce any kind of relevant inflammasome activation in podocytes even with genetic overactivation with the Muckle-Wells genetic variant, which is known to be functional in mice. Our findings contrast many published reports on NLRP3 inflammasome activity in podocytes and may raise doubts about published data in other epithelial cells. A careful assessment and independent validation with suitable experimental and analytical tools may help to solve these discrepancies.

## Data availability statement

Data and materials availability: scRNAseq data are available in the National Center for Biotechnology Information’s Gene Expression Omnibus repository with accession numbers GSE195797 and GSE212273.

## Ethics statement

Ethical approval was not required for the study involving humans in accordance with the local legislation and institutional requirements. Written informed consent to participate in this study was not required from the participants or the participants’ legal guardians/next of kin in accordance with the national legislation and the institutional requirements. The animal study was approved by Regierung von Oberbayern (ROB). The study was conducted in accordance with the local legislation and institutional requirements.

## Author contributions

SK: design and conduction of *in vivo* and *in vitro* experiments, data analysis, and manuscript preparation; JM: design and conduction of *in vivo* and *in vitro* experiments, data analysis, and manuscript preparation; MK: Deep-learning-assisted morphometry; TH: *in vivo* and *in vitro* experiments; CL: RNAseq data analysis; MM: *in vivo* and *in vitro* experiments; CW: histological analysis; LC and RS: scRNAseq data analysis; MA and GA: STED imaging and analysis; MEM: manuscript preparation; PN: supervision; H-JA: experimental design, data analysis, funding, and manuscript preparation. All authors contributed to the article and approved the submitted version.
