# Supplementary material for: No NLRP3 inflammasome activity in kidney epithelial cells, not even when the NLRP3-A350V Muckle-Wells variant is expressed in podocytes of diabetic mice
Source: Front Immunol. 2023 Aug 23;14:1230050. doi: 10.3389/fimmu.2023.1230050 (PMC10513077; doi:10.3389/fimmu.2023.1230050)
Supplement: Supplementary file 1 [file DataSheet_1.pdf]

No NLRP3 inflammasome activity in kidney epithelial cells not even when the NLRP3-A350V Muckle-Wells variant is expressed in podocytes of diabetic mice

Sophie Carina Kunte*, Julian A. Marschner[1]*, Martin Klaus[1], Tâmisa Honda[1], Chenyu Li[1], Manga Motrapu[1], Christoph Walz[2], Maria Lucia Angelotti[3], Giulia Antonelli[3,4], Maria Elena Melica[3], Letizia De Chiara[3], Roberto Semeraro[5], Peter J. Nelson[1], Hans-Joachim Anders[1]

1 Nephrologisches Zentrum, Medizinische Klinik und Poliklinik IV, Klinikum der Universität München, LMU München, Germany

2 Pathologisches Institut der LMU, Klinikum der Universität München, LMU München, Germany

3 Department of Experimental and Biomedical Sciences "Mario Serio" University of Florence, Florence, Italy

4 Nephrology and Dialysis Unit, Meyer Children's Hospital IRCCS, Florence 50139, Italy

5 Department of Experimental and Clinical Medicine, University of Florence, Florence, Italy.

Supplementary material

Supplementary table 1: RPC culture medium composition..
Supplementary table 2: PCR reaction mixtures..
Supplementary table 3: Primer pairs used for qPCR and genotpying..
Supplementary table 4: Narcosis, antagonization and analgesia..
Supplementary table 5: Antibodies and reagents for immunohistochemical and immunofluorescence staining..
Supplementary table 6: Reagents and kits..
Supplementary figure 1: Genetic model of Nphs2-Cre; Nlrp3WT/A350V mice..
Supplementary figure 2: Phenotypic comparison of three different STZ treatment regimes in male and female C57BL/6J mice..
Supplementary figure 3: Unbiased single cell RNA sequencing of healthy murine kidneys indicate absence of canonical NLRP3 inflammasome..
Supplementary figure 4: Deep learning strategy for glomerular and podocyte morphometry in WT-1 and nephrin stained immunohistochemistry slides..
Supplementary figure 5: BUN..

Supplementary table 1: RPC culture medium composition

Reagent	Company	Cat number	Volume
Endothelial cell growth medium	PromoCell	C-39210	400 ml
Hyclone fetal bovine serum defined	Cytiva	SH30070.03	100ml
DMEM-F12	Sigma Aldrich	D2906	15.6g/L
Panobinostat	Selleckchem	S1030	100nM
Penicillin/ streptomycin	PAN Biotech GmbH	P06-07100	5ml

Supplementary table 2: PCR reaction mixtures

Reagent	Amount per reaction [µl]	Company	Cat number
Reverse transcriptase PCR			
dNTP Set (25 mM per base)	0.45	Thermo Fisher Scientific	R0186
DTT 0,1 M	1	Thermo Fisher Scientific	18080085
5x First Strand Buffer	4.5	Thermo Fisher Scientific	18080085
Hexanucleotide Mix	0.25	Roche Diagnostics	11277081001
Linear Acrylamide (15 µg/ml)	0.25	Thermo Fisher Scientific	AM9520
RNasin R Ribonuclease Inhibitor	0.5	Promega	N2515
SuperScript TM III Reverse Transcriptase or ddH2O	0.5	Thermo Fisher Scientific	18080085
Quantitative real-time PCR			
BioStab PCR Optimizer	4	Biomol	62508.5
Bovine Serum Albumin PCR grade	0.2	Thermo Fisher Scientific	B14
ddH2O PCR grade	1.2	Invitrogen	10977035
dNTP Set (25 mM per base)	0.15	Thermo Fisher Scientific	R0186
MgCl 2 25mM	2.4	Thermo Fisher Scientific	R0971
Primer forward 10µM	0.6	Metabion international	-
Primer reverse 10µM	0.6	Metabion international	-
SYBR R Green I nucleic acid gel stain	0.04	Sigma-Aldrich Chemie	86205
Taq DNA Polymerase	0.16	New England Biolab	M0273X
10X Taq Buffer without Detergent	2	Thermo Fisher Scientific	B55

Supplementary table 3: Primer pairs used for qPCR and genotpying

Target gene	NCBI key	Forward primer (5‘ → 3‘)	Reverse primer (5‘ → 3‘)
Nphs1	NM_004646.4	GTCTGCACTGTCGATGCCAATC	CCAGTTTGGCATGGTGAATCCG
Nphs2	NM_014625.4	CTGTGAGTGGCTTCTTGTCCTC	CCTTTGGCTCTTCCAGGAAGCA
Nph2-Cre	NA	AGGTTCGTTCACTCATGGA	TCGACCAGTTTAGTTACCC
Nlrp3^{A350}	NA	GCTACTTCCATTTGTCACGTCC	CGTGTAGCGACTGTTGAGGT

v		and CACCCTGCATTTTGTTGTTG	
IL2	NC_000069.7	CTAGGCCACAGAATTGAAAGATCT	GTAGGTGGAAATTCTAGCATCATCC
Rn18s	NR 003278.3	GCAATTATTCCCCATGAACG	AGGGCCTCACTAAACCATCC

Supplementary table 4: Narcosis, antagonization and analgesia

Drug	Concentration [mg/kg BW]	Treatment regime	Cat number	Company
		Narcosis		
Medetomidine	0.5	i.p. injection, once prior to surgery, surgical tolerance verified by toe pinching	07725752	Zoetis
Midazolam	5		4921530	ratiopharm
Fentanyl	0.05		2084366	Janssen-Cilag
		Antagonization		
Atipamezol	5	s.c. injection, once	8-00732	CP-Pharma
Flumazenil	0.1		4470990	Hexal
		Analgesia		
Buprenophin	0.1	i.p. injection, once 30min prior to antagonization, then every 8h for 3 consecutive days	01498870	Bayer Vital
Metamizol-Natrium 1H2O	200	p.o. application, 5min prior to narcosis induction	0731672	Sanofi-Aventis

Supplementary table 5: Antibodies and reagents for immunohistochemical and immunofluorescence staining

Antibody for antigen (rabbit anti-mouse)	Target/ host	Secondary antibody/ avidin-biotin complex	Dilution/ incubation time	Cat number	Company
		Primary antibodies			
WT-1	Mouse/ mouse	Biotinylated anti-rabbit IgG/ VECTASTAIN	1:400 (PBS) over night 4°C	sc-393498	Santa Cruz Biotechnology
Nephrin	Mouse/ guinea pig	Biotinylated goat anti-guinea pig IgG/ VECTASTAIN	1:800 (10% milk, PBS) over night 4°C	BP5030	Origene

Nlrp3	Mouse/ mouse	Biotinylated goat anti-rabbit IgG2a/ VECTASTAIN	1:1000 (10% milk, PBS)/ over night 4°C	TA323326	OriGene
		Secondary antibodies			
IgG (H+L), biotinylated	Rabbit/ goat	-	NLRP3: 1:300 (10% milk, PBS) WT1/ Nephrin: 1:1000 (PBS)/ 30min RT	ZRB1001	Linaris
		Avidin-biotin complex/ substrate			
VECTASTAIN® Elite® ABC-HRP Kit	-	-	-	PK6100	Vector Laboratories

Supplementary table 6: Reagents and kits

Reagent	Company	Cat number
Breeder Brinsea Octagon 20 Advance	J.Hemel Brutgerät	32150
Pure Link RNA Mini Kit	Invitrogen	12183018A
RET-3-ISO Isolated Rectal Probe for Mice	Physitemp Instruments	NA
RNAlater	Ambion,	AM7021
Rodent surgery table (heatable)	Medax	M12511
Suture Ethibond Excel 5-0	Ethicon	EH7260H
Suture Vicryl TM 5-0	Ethicon	V493H
Thermes-USB Temperature Data Acquisition Seven channel unit	Physitemp Instruments	NA

Supplementary figure 1: Genetic model of Nphs2-Cre; Nlrp3WT/A350V mice.

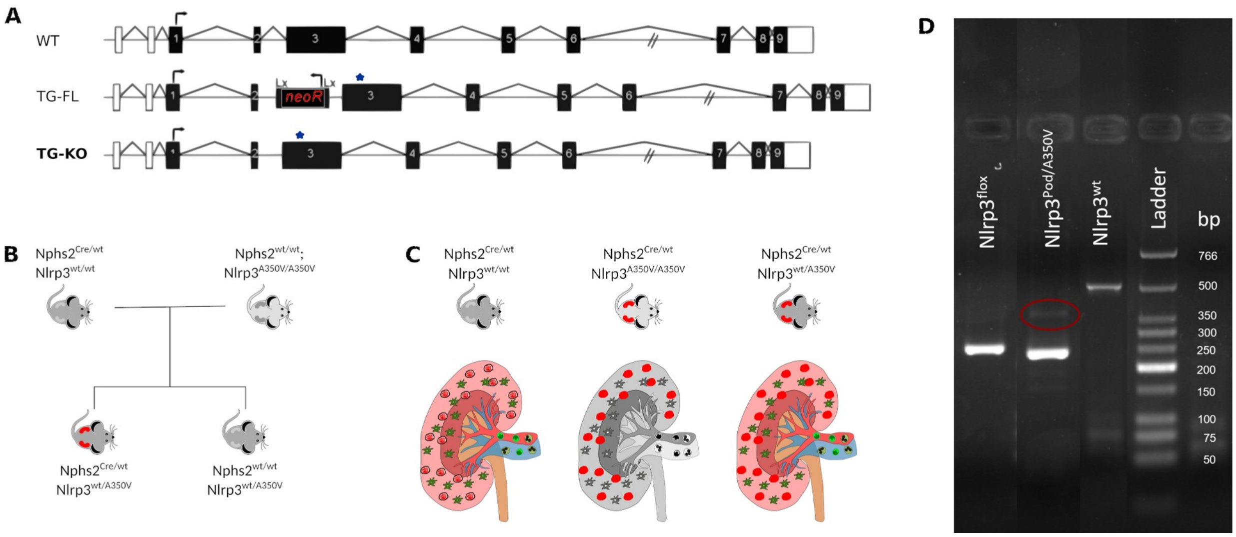

(A) Wildtype *Nlrp3* allele (WT, top), modified *Nlrp3* allele with the knocked-in floxed neoR cassette and point mutation in exon 3 (transgene-flox, TG-FL, middle) and the same construct after Cre-mediated excision (transgen-knockout, TG-KO, bottom) of the murine Nlrp3 allele. **(B)** Breeding strategy to produce Nphs2-Cre; Nlrp3$^{WT/A350V}$ (i.e. Nphs2$^{Cre/WT}$; Nlrp3$^{WT/A350V}$) and Nlrp3$^{WT/A350V}$ (i.e. Nphs2$^{WT/WT}$; Nlrp3$^{WT/A350V}$) littermates for experimentation. **(C)** Homozygous Nlrp3$^{A350V/A350V}$ mice (middle) are effectively Nlrp3 whole body knockouts, as the neoR cassette permits functional Nlrp3 transcription, unless the cassette is cut out by a Cre recombinase. In this study, we deployed mice carrying only one copy of the TG-FL allele, so that the NLRP3 inflammasome remains functional throughout the body, but is overactive in podocytes only. **(D)** End point PCR with primer pairs as suggested by The Jackson laboratories for the Nlrp3^{A350V} allele on isolated glomeruli from Nlrp3$^{A350V/A350V}$ (left lane), Nphs2-Cre; Nlrp3$^{WT/A350V}$ (middle lane), and Nlrp3$^{WT/WT}$ (right lane) mice, which suggests, that the shorter, floxed-out product was amplified in Nphs2-Cre; Nlrp3$^{WT/A350V}$ mice (red circle).

Supplementary figure 2: Phenotypic comparison of three different STZ treatment regimes in male and female C57BL/6J mice.

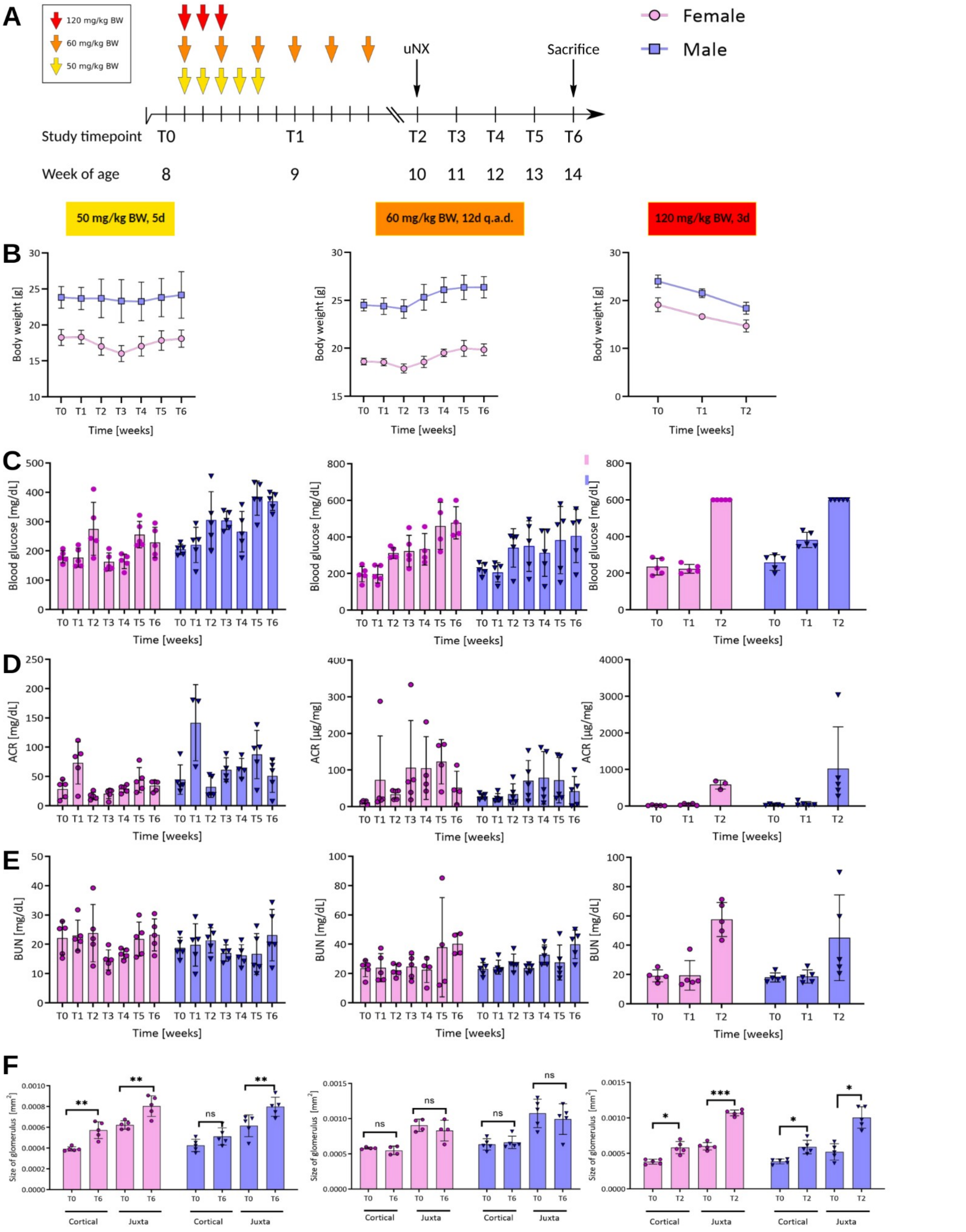

(A) Prior to uNX male and female C57BL6/J mice were injected i.p. with STZ as follows: 120 mg/kg BW for 3 consecutive days (red), 60 mg/kg BW, every other day for a total of 6 injections (orange), 50 mg/kg BW for 5 consecutive days (yellow). Mice were followed for 4 weeks after uNX. Time course of body weight **(B)**, blood glucose **(C)**, ACR **(D)**, and BUN **(E)**. **(F)** Morphometric determination of glomerular size of STZ/ uNX treated mice from PAS stained paraffin sections; n = 5, * $p<0.05$, **** $p < 0.0001$.

Supplementary figure 3: Unbiased single cell RNA sequencing of healthy murine kidneys indicate absence of canonical NLRP3 inflammasome.

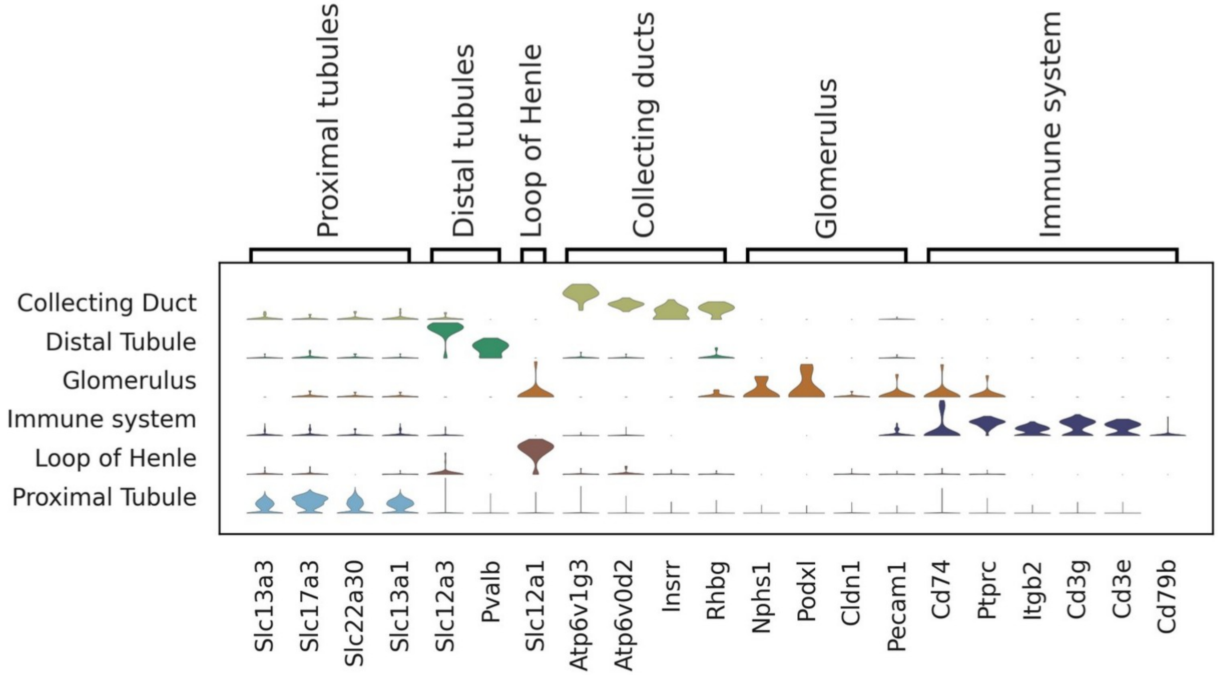

Expression pattern of selected marker genes (x-axis) used for Uniform Manifold Approximation and Projection (UMAP) cluster annotation shown in figure 1B.

Supplementary figure 4: Deep learning strategy for glomerular and podocyte morphometry in WT-1 and nephrin stained immunohistochemistry slides

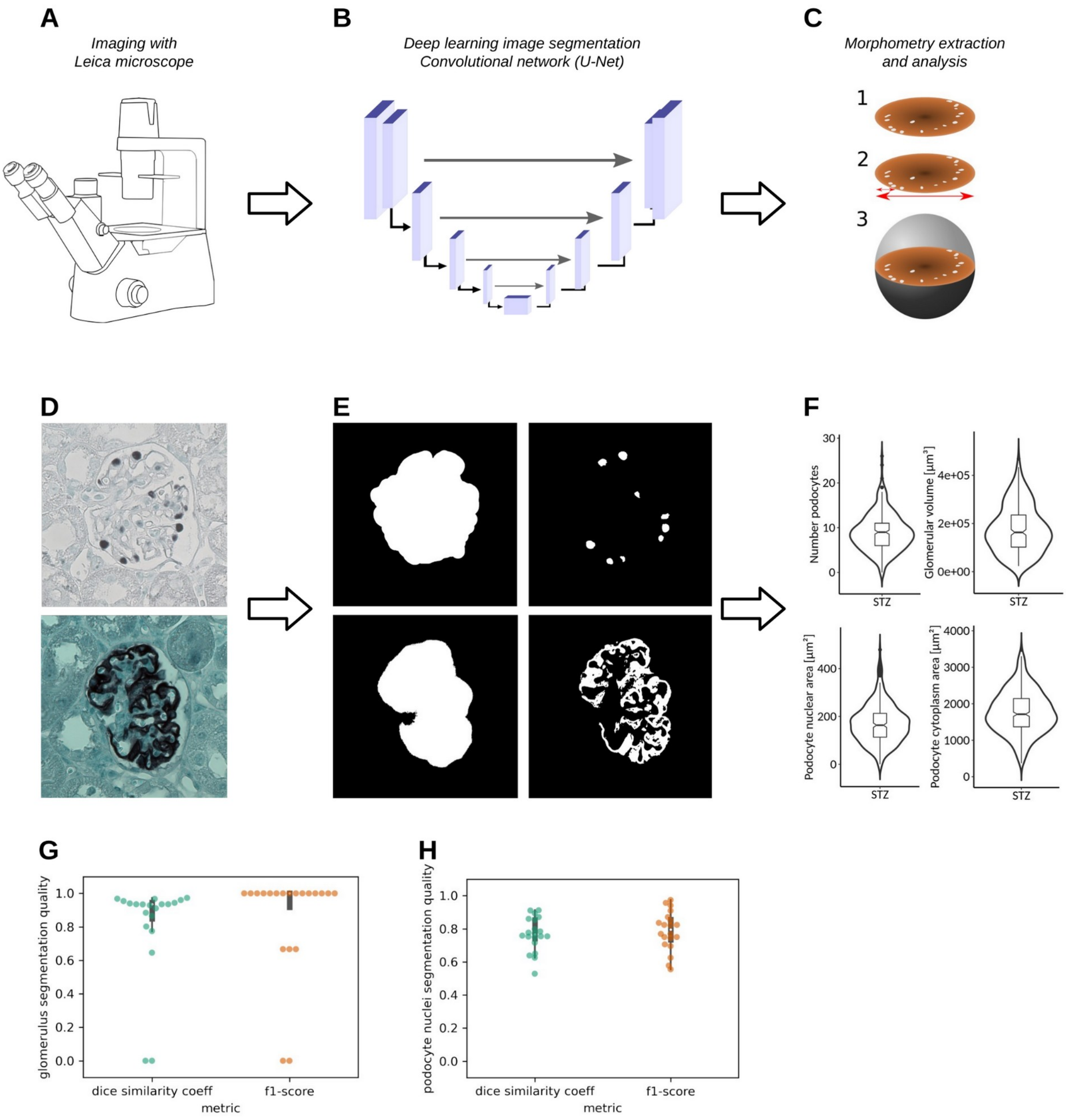

The general deep leanining morphometry pipeline is presented in **(A-C)**: Histology slides are imaged and digitalized using a microscope **(A)**. A U-Net deep learning segmentation algorithm is implemented and trained to segment structures of interest in a supervised manner **(B)**. Obtained segementations are used to perform morphometric analysis **(C)**. In this study, WT-1 and nephrin

stained glomeruli were imaged using Leica DMRBE Research Microscope and saved in 2048x2048 pixel frames with resolution 8.8 pixels per micron. Each file contains at least one glomerulus **(D)**. Images are resized to 1024x1024 pixel, fed into the U-net segmentation network, and glomerular tufts or podocyte nuclei (WT-1 staining) are segmented. The podocyte cytoplasm (nephrin staining) is obtained via Otsu thresholding from the glomerular tuft segmentations **(E)**. The segmentations are used to obtain the morphometric analysis of glomeruli and podocytes **(F)**. Deep learning segmentation quality of the glomerular and the podocyte nuclei segmentation is assesed using the common metrics dice similarity coefficient and f1-score **(G, H)**.

Supplementary figure 5: BUN

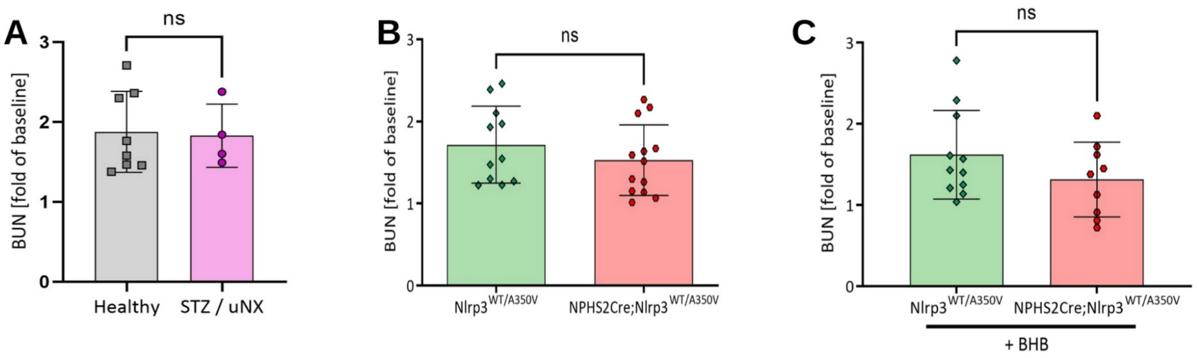

BUN measurements at T6 **(A)** and T12 **(B,C)** as part of the phenotyping shown in main figures 4 **(A)**, 5 and 6 **(B)**, and 7 **(C)**.
